# Microplastics removal from aqueous environment by metal organic frameworks

**DOI:** 10.1186/s13065-023-01032-y

**Published:** 2023-09-21

**Authors:** Zhila Honarmandrad, Massoud Kaykhaii, Jacek Gębicki

**Affiliations:** Department of Process Engineering and Chemical Technology, Faculty of Chemistry, Gdańsk University of Technology, Narutowicza 11/12, Gdańsk, 80-233 Poland

**Keywords:** Microplastics, Metal organic frameworks, Adsorption, Water treatment technology

## Abstract

This paper provides an overview of recent research performed on the applications of metal–organic frameworks (MOFs) for microplastics (MPs) removal from aqueous environments. MPs pollution has become a major environmental concern due to its negative impacts on aquatic ecosystems and human health. Therefore, developing effective and sustainable methods for removing them from aqueous environments is crucial. In recent years, MOFs have emerged as a promising solution for this purpose due to their unique properties such as high surface area, renewability, chemical stability, and versatility. Moreover, their specific properties such as their pore size and chemical composition can be tailored to enhance their efficiency in removing MPs. It has been shown that MOFs can effectively adsorb MPs from aqueous media in the range of 70–99.9%. Besides some high price concerns, the main drawback of using MOFs is their powder form which can pose challenges due to their instability. This can be addressed by supporting MOFs on other substrates such as aerogels or foams. Meanwhile, there is a need for more research to investigate the long-term stability of MOFs in aqueous environments and developing efficient regeneration methods for their repeated use.

**Supplementary Information:**

The online version contains supplementary material available at 10.1186/s13065-023-01032-y.

## Background

Production of plastics has increased dramatically in the recent decades and reached 0.3 billion metric tons per year, of which half is for single-use [1]. It is estimated that over 5 Gt of plastic waste is globally scattered in the environment. In 2021, about 390.7 million metric tons of plastics are manufactured from which, about 13 million tons were entered into rivers and by the end of 2025, it is estimated that 250 million tons of plastics will be released to waters [2]. There are 25,000 trade names for plastics with 15,000 variants and they have 30–40 types. They are obtained from coal and oil and consist of one or more long chains of carbon as their molecular structure which is bonded with elements such as hydrogen, oxygen, nitrogen, chlorine and sulfur. High-density polyethylene (HDPE), polyvinyl chloride (PVC), polystyrene (thermocole, PS), polypropylene (PP) and polyethersulfone (PES) are the widest use plastics, accounting for about 75% of plastics production. They are mainly used in packaging, electronics, automotive manufacturing and construction. In the environment, factors such as physical abrasion, sunlight, weathering, and biological degradation can fragment plastics to smaller particles, in part into hazardous microplastics (MPs) [3]. MPs are identified as plastic fragments with a size of several millimeters, typically less than 5 mm [4]. MPs has a range of sizes and variety in shapes with different compositions and include aliphatic or aromatic structures with various functional groups and may contain dyes, blends and copolymers. Wind transfers MPs easily for hundreds of miles and they can also be transported over great distances via ocean currents [5]. They can also be carried out from land into waterways through rainfall and sink in rivers and oceans [6]. Besides industries and factories which manufacture them, direct throwing out disposable plastics, laundering of synthetic clothes, microbeads from cosmetic products, scrape of automobile tires, and fragmentation of large particles of plastics are another most important sources of entrance of MPs to the aquatic streams [7, 8]. It is estimated that quantity of MPs which enters into seawater is between 4.8 and 12.7 million metric tons [9], while already around 14 million metric tons MPs are deposited on the floor of the oceans [10]. Because of the floating character of plastic particles, they can easily be spread around the water, passing them to gather on seashores, where they may remain for millions of years. MPs have been found everywhere, from the earth’s atmosphere to the sediments of deep seas, from ice in the poles to all ecosystems. Franeker, et al. [11] studied the stomachs of 1295 of seabirds during the years 2003–2007 and found that 95% of them contained an average of 34.5 ± 2.5 pieces of MPs with an average mass of 0.3 ± 0.02 g. Recently, it has been shown that MPs can be absorbed by the roots of plants and translocated to aerial tissues and can be accumulated in their organs [12]. They can also remain in the terrestrial and sea plants [13]. It is proved that MPs can even have a significant effect on microorganisms during fermentation of biomass [14].

Due to their high ratio of surface area to mass, MPs can adsorb bacteria and pollutants and toxins such as pharmaceuticals, heavy metals, personal care products and so on which can be ingested by aquatic animals and finally be consumed by humans posing an unpredictable health risk [15, 16]. MPs can also enter into the human bodies through contaminated drinks, foods and breathing [17], as a result, MP particles have been seen in human blood and stool [18]. Recently, MPs have been declared as contaminants of emerging concern and present a significant risk to human health as endocrine disruptors [19, 20]. Consequently, MPs can cause cancer, malformation in animals and humans, impaired reproductive activity, and reduce immune response [21]. MP fibers in the respiratory system can cause tumors in the lung and azo dyes used for polymers colouring may cause endocrine disruption, dermatitis, and hormone dysregulation [22]. It should be mentioned that toxicity of MPs is not only because of their own nature but also is due to the various additives and plasticizers that are added to them during their manufacturing [23, 24]. Khan & Jia recently summarized the impact of MPs over ecosystems and human health [25]. The European Union started a strategy on controlling release of MPs into waterways to reduce their pollution in water resources [26].

## Removal methods of MPs from aqueous environment

**Fig. 1:**
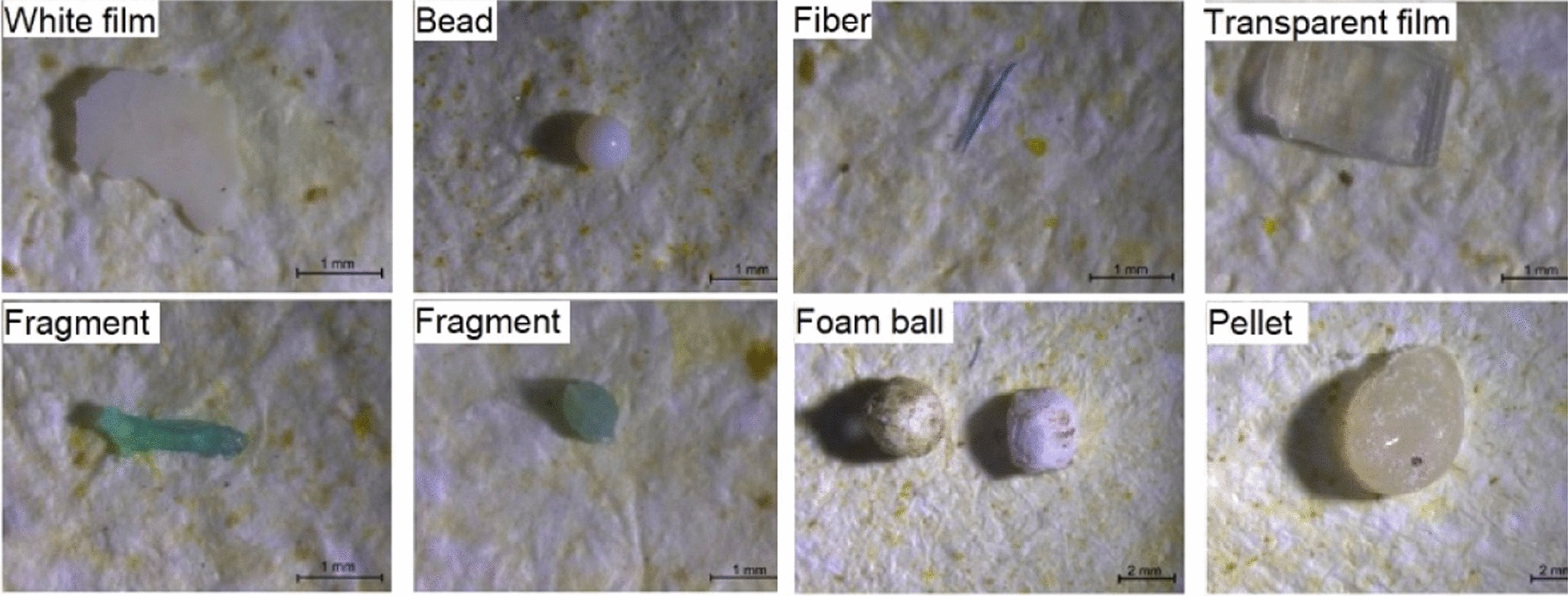
Main types of MPs present in water. They have hybrid characteristics with different sizes, shapes, abundance, densities, and appearance (From [35] with kind permission of the copyright owner)

To keep all of the sources of water safe, there is a need to remove MPs as a major water pollutant, which is also a major concern globally. On the other hand, one of the most important ways that MPs can enter the human body is through drinking contaminated water. Sewage/ WWTPs cannot completely remove MPs from water and wastewater. It has been shown that even after treatment, about 73.8% of MPs can be accumulated in the sludge of the WWTPs [27], so, the effluent released from these plants contains substantial quantities of MPs [28]. If these effluents mix with freshwater, MPs enter the drinking water supply chain [29]. Another source for MPs entering the water distribution systems is via the system itself, since many parts of water treatment plants and water distribution and pipeline systems are usually made up of polymers such as PP, HDPE, and PVC [30]. MPs in the water resources are mainly found in the forms of fibers, fragments, pellets, foams and films (Fig. 1) [31]. The obvious harmful effects of MPs on the environment, human health, and ecology, led scientists to investigate ways to remove them from aquatic media [32].

During treatment in WWTPs, water passes through three levels of primary, secondary, and tertiary treatments [33]. Sometimes, there is also an initial step for removal of large floating objects. Throughout primary treatment, insoluble solids from the wastewater physically settle down. At this stage, 35–59% of MPs can be removed by their trapping in solid flocs and screening the light floating MPs. This process leads to a 37% increase in small particles (0.1–0.5 mm). The removal efficiency in water and WWTPs is calculated based on the MPs concentration in the influent and effluent, i.e. the number of MPs present per liter of the liquid. By using the attached growth or suspended growth system, during the secondary (biological) treatment stage, targets degradation of the biological content of the wastewater happens. At this stage, 50–98% of MPs can be removed. The main mechanism of MPs separation at this stage is the skimming and settling of the entrapped MPs by gravity. It is proven that this treatment removes fragments more effectively than microfibers. Tertiary (advanced) stage of the treatment process is the final stage in which chemical disinfection, ozonation, reverse osmosis, ultra/micro/nano-filtration, and other advanced techniques are utilized. For tertiary treatment, different mechanisms are suggested for the removal of MPs. Therefore, efficiency of this stage for the removal of MPs is highly dependent on the technologies or processes that are employed. For example, chemical processes are involved in advanced oxidation treatment processes while physical separation is the main mechanism in filtration technology. In comparison with membrane filters, rapid sand and continuous backwash filters release more microbeads [34, 35]. Additional file 1: Table S1 depicts the conventional methods for removal of MPs from aqueous media [34]. Some of these conventional techniques are further modified for MPs removal. It is proved that MPs removal can be reached to 88% in the absence of tertiary treatment step and up to 97% with this treatment [36]. By combining various treatment technologies, modern WWTPs can remove significant amounts of MPs (88–99%). However, less than 2% of MPs can still escape from WWTP and reach the aquatic ecosystem, which can be mixed with our drinking water [22]. Moreover, MPs that remain in sludge receive no further treatment and often are directly disposed of on land. The consequences is contamination of soil and groundwater through percolation [37]. In many instances, groundwaters are used for drinking.

It seems that there is no special technology for total MPs removal in conventional WWTPs. As a result, researchers tried to find techniques to separate them from aqueous media by employing filtration, (electro)coagulation, foam/froth flotation, magnetic extraction/separation, membrane separation technology, agglomeration, density separation, adsorption removal, oil film separation, and advanced oxidation processes [38]. There are review articles which discuss in detail these techniques that can be used for remediation of MPs [34, 35]. Below, the most important of them are explained briefly.

It is possible to remove MPs from wastewater during ultrafiltration (2–100 μm) [39]. Similar to any other membrane-based filtration, size exclusion acts as the main removal mechanism. It has been proved [40] that this method has the highest efficiency for the PE, PVC and PES removal with efficiency of > 90% with a dominant size range of 100–190 μm. The main drawback with this technique generally is the fact that it requires a proper chemical pretreatment and suffers from membrane fouling [41]. Granular medium, flow conditions and solution properties are the other parameters which can influence the separation efficiency [42]; however, it is difficult to elute the near-sieve particles from blocked filters. Thick layers of cake and larger PE particles are ideal for the fouling resistance of membranes. In the presence of large PE particles, the flux of membranes decreased by only 10%. By introduction of membrane bioreactor technology which combines membrane process with a biological catalysts process a high MPs retention up to 99.9% reported [43, 44]. But it is a long, sophisticated technique.

Coagulation is one of the most efficient techniques for removal of solid suspension, especially hydrophobic substances during wastewater treatment from aqueous media and hence it has also been verified as a promising process for MPs removal from wastewater. It was demonstrated that aluminium and iron salts can remove MPs up to 36.89% for particle sizes < 0.5 mM [45]. Coagulation is generally better for removal of MPs with smaller sizes. One main problem with coagulation is the large amount of coagulants and polymeric additives that remain in the effluents due to their large quantities usage during the coagulation process [46]. Moreover, during the weathering processes, 1–5 μm MPs particles can be escaped which has a negative effect on removal efficiency. In fact, coagulation works better for MPs removal when it is combined with the other physical separation processes such as filtration, membrane separation, and froth flotation [47]. However, regarding remediation of MPs, presence of large amounts of coagulants can block out the surface and pores of membranes. In contrast to coagulation, in electrocoagulation coagulants are generated electrically, as a result, in electrocoagulation there is no need for chemicals, which makes it more environmentally friendly. Usually, metal hydroxide coagulants are generated by the reaction between Fe^2+^ and Al^3+^ ions released from the metal electrodes and the OH¯ ions which are produced after electrolysis. In the presence of these coagulants, MPs particles become destabilized and subsequently entrap in the sludge blanket made by the coagulants, which are then removed from the media [48]. For colloidal stability, similar to coagulation, in agglomeration the surface properties of MPs is the most important parameter. However, this parameter is rarely considered in the current researches. Colloids stability in water depends on the contributions of van der Waals and repulsive electrostatic forces, which is not always possible [49].

The process in which MP particles are trapped in the sludge blanket as a result of coagulation, is called pulse clarification. Sarkar et al. found that filtration and pulse clarification have a potential of removing 85% of MPs [50]. The pulsation has the effect of preventing formation of sludge blanket from contracting, hence, reducing the amount of entrapment.

Density separation is another way to isolate MPs from sediments. After stirring, MPs which are lighter than media float to the upper layer of suspension and can be easily separated from sediments. However, for the density separation, an adjustable density media for flotation is necessary. Despite the excellent applicability, density flotation only applied for laboratory scale samples so far [51].

**Fig. 2:**
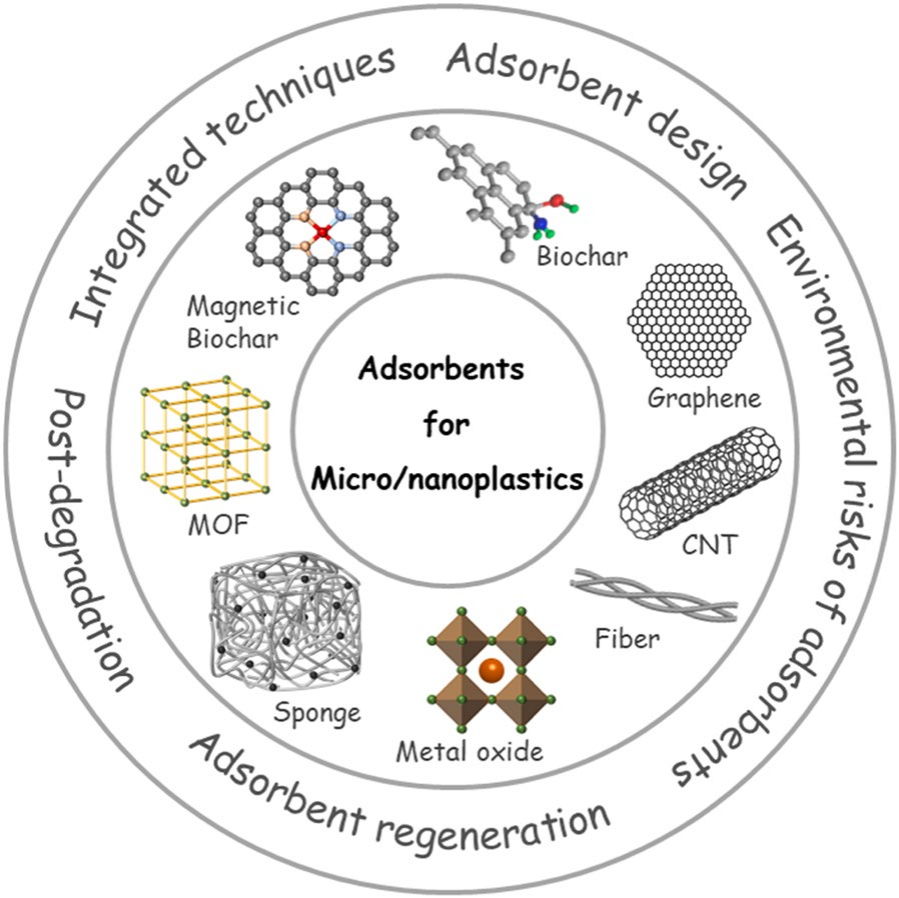
Different engineered adsorbents that are commonly in use for the removal of MPs/NPs from contaminated water. Besides design of adsorbents, other challenges in this field are their regeneration, environmental risk, and post degradation (From [53] with kind permission of the copyright owner)

Adsorption is another conventionally used water treatment process which is widely used because of its simplicity, high efficiency, and being economical. The main advantage of adsorption is the possibility of selecting a wide range of adsorbing materials that have a higher affinity towards the desired molecules [52]. Diverse adsorbents have been developed for the adsorption and removal of pollutants from water resources in the last few years, including biochar, protein sponges, metal oxides/hydroxides, and metal–organic frameworks (MOFs) [53]. Nanomaterials such as MOFs are known as excellent adsorbents due to their huge surface areas, ease of synthesis and rapid functionalization, and high association with various pollutants. Some recent review articles described the role of adsorption in the remediation of MPs [54–56]; meanwhile, some other reviews discuss the various adsorbents for treatment of MPs contaminated water [53, 57–59]. Figure 2 presents different engineered adsorbents that are commonly in use for the adsorption of MPs from aqueous media [53]. It has been shown that removal efficiency of these adsorbents is between 25% for biochar [60] to ~ 100% for zinc laurate-TiO_2_ particle coating [61]. It should be noted that the adsorption capacity of adsorbents is highly affected by the shape and size of MPs. Due to their smooth and edge-less surfaces, microbeads are adsorbed to a lesser extent compared to the MPs with irregular shapes [62]. As a result, it cannot be concluded that which adsorbent acts as the best for MPs removal in general, since it depends mainly on the MPs understudy.

As mentioned earlier, MOFs are under increasing attention in water remediation due to their successful applicability [38]. These materials are classified as highly ordered crystalline metal clusters with very high porosity (> 90%) which are composed of metal–oxide clusters and organic linkers [63]. They have extremely large surface area (up to 10,000 m^2^/g [64]), which make them suitable for a wide variety of applications [65–67], including MPs adsorption [68]. By changing metal oxides and organic linkers, it is possible to control the pore size, volume, and functionality of MOFs to adjust them for any designable applications. Also, the characteristics of MOFs mainly depend on the nature of the selected inorganic and organic nodes and ligands and their connectivity [69]. MOFs are also characterized by their ease of synthesis and modification [70]. These features make them an excellent candidate for water treatment applications besides being widely employed in the adsorption of inorganic and organic pollutants from water, owing to their stable structures and their high adsorption capacity [71]. They showed high performance in recycling and large-quantity filtration experiments as well. The functional groups of MOF can form hydrogen bonding or van der Waals interaction with MPs. The positively charged defects present in the MOF show high affinity to a wide range of negatively charged MPs [72].

In the following sections, applications of MOF-based adsorbents for removing MPs from aqueous environments are presented.

### Application of MOFs for removal of MPs from water and wastewater

As mentioned, a range of techniques have been proposed for the removal of MPs from water and wastewater, however, these methods can be expensive and may not be effective in removing MPs, especially smaller particles [72]. Recently, new methods based on adsorbents such as MOFs are presented for removing MPs from an aqueous environment [73]. One of the significant advantages of using MOFs for MP removal is their high selectivity toward these particles. MOFs can be tailored for specific applications by modifying their composition and pore size to selectively capture different sizes and types of MPs. This selectivity allows more efficient removal of MPs while minimizing the impact on other water constituents. Application of MOFs for the removal of MPs from water and wastewater is a promising method which offers a range of advantages over other techniques. Table 1 shows the advantages of using MOF in MPs removal and its comparison with other methods. As can be seen, using MOF has several advantages over other methods in terms of efficiency, selectivity, regenerability, scalability, and environmental impact. They can selectively adsorb microplastics while leaving other materials in the water untouched. MOFs can also be regenerated and reused for multiple cycles, making them more cost-effective and sustainable compared to single-use filters or other methods that require frequent replacement. MOFs are made from non-toxic materials and do not produce harmful byproducts during the purification process, making them a more environmentally friendly option compared to some other methods. In contrast, filtration can remove microplastics but may not be as selective as MOF, and filters need to be replaced frequently. Sedimentation can be less effective for MPs removal. Chemical treatments can be highly effective but can produce hazardous waste and harm the environment [35, 73–76].

**Table 1:** Advantages of using MOF for MPs removal in comparison with the other methods

Benefit	MOF	Filtration	Sedimentation	Chemical treatments
Efficiency	High	Medium to high	Low to medium	High
Selectivity	High	Low to medium	Low to medium	High
Regenerability	Yes	No	No	No
Scalability	High	Medium	Low	Medium to high
Environmental	Low impact	Low impact	Medium impact	High impact

As research in this area continues to progress, we can expect to see the development of more efficient, cost-effective, and sustainable methods for MPs removal. Several research studies have explored the use of MOFs for the removal of MPs from water and wastewater [34, 71]. In this first review, we critically reviewed advantages and weaknesses of using different MOFs. An attempt has been made to collect and analyze all available research studies on this subject. Table 2 summarizes applications of MOFs in the removal of MPs.

**Table 2:** Summary of the use of MOFs in the removal of MPs

MOF	MPs	Size of MPs	Media	Removal efficiency (%)	Refs.
2D MOF@C@FeO	NM	1000 nm	Water	100	[19]
Nano-Fe@ZIF-8	PS	1.1 μm	Water	≥ 98	[20]
ZIF-67	PSMPs	1.0 to 3.0 μm	Water	92.1	[38]
UiO-66-X	PVDF, PMMA and PS	273 nm	Water	95.5	[72]
MIL-100 (Fe)	PSF, PVC	40 μm (PE) and 140 μm (PVC)	Water	NM	[77]
ED- MIL 101(Cr) UF	NM	NM	Wastewater	90	[78]
Ag_2_O/Fe-MOF	PEG, PE, PET	NM	Deionized water	NM	[80]
ZIF-8@Aerogel	PVDF, PS	60–110 nm (PVDF) and 90–140 nm (PS)	Water	91.4 (PVDF) − 85.8 (PS)	[81]

^a^*NM* Not mentioned

In a study, Chen et al. [72] loaded melamine foam with Zr-based UiO-66-X MOFs, which were created using the 1,4-dicarboxybenzene ligand with different functional groups. The researchers tested the MOF-based foam’s ability to remove MPs from a simulated suspension of three different types of plastics: PVDF, PMMA, and PS. The results showed that the MOF-based foam was able to achieve a removal rate of up to 95.5% +1.2%, with the removal rate affected by the particle size and zeta potential of the MPs. The MOF-based foam was also found to have good stability and reusability in repeated adsorption-desorption cycles, meaning that it could be used multiple times to remove MPs from water, making it a cost-effective solution for MP pollution. Furthermore, the researchers investigated the effect of MOF loading and foam density on the adsorption capacity of the MOF-based foam. They found that increasing the MOF loading and foam density resulted in higher adsorption capacity, indicating that more MOF material and higher foam density could improve the ability of the foam to remove MPs from water. It is worth noting that smaller MPs were more difficult to remove than larger ones due to their increased mobility in water, which makes them more challenging to capture. Nonetheless, the study demonstrated the key benefits of MOFs for removing MPs, including their high porosity, ability to trap pollutants, and good durability. In summary, MOF-based foams show promise as a practical solution for MP pollution in water. The high adsorption capacity, stability, and reusability of the MOF-based foam make it a promising approach for MP removal, and further research is needed to optimize the foam design and assess their performance in real-world water treatment applications.

Gnanasekaran et al. [77] conducted a study in which they investigated the effectiveness of a MOF membrane called MIL-100 (Fe) for removing MPs from textile wastewater. MIL-100 (Fe) is a type of MOF that contains iron metal ions and terephthalic acid organic ligands, resulting in a mesoporous structure with a small pore diameter (1.8 nm), large pore volume (0.8374 cm^3^/g) and microporous windows which allow for the transport of small molecules.

To create the blended membrane, the researchers integrated hydrophilic MIL-100 (Fe) nanoparticles into a polysulfone matrix. The incorporation of MIL-100 (Fe) significantly affected the morphology of the membrane, including its hydrophilicity, wetting energy, work of adhesion, porosity, and pore size. The best performance was achieved using a 0.5 wt% loading of MIL-100 (Fe) in the polysulfone matrix (M0.5), which provided a 10.3-fold increase in pure water flow compared to the pristine polysulfone membrane (M0). The PSF/MIL-100 (Fe) membrane showed optimal performance in an alkaline environment (pH 9) due to the electrostatic repulsion mechanism towards cationic-charged contaminants. The study also demonstrated the membrane’s reusability, as the organic ligands in the MIL-100 (Fe) structure help to retain its stability even after several cycles of use.

Overall, the results of this study suggest that the PSF/MIL-100 (Fe) blended membrane is an effective method for removing MPs from textile wastewater. The use of MIL-100 (Fe) in the membrane design contributes to its high surface area, which is critical for efficient removal of MPs. The researchers noted that further studies are needed to optimize the membrane design and operating conditions to improve its performance and efficiency.

Mohana et al. [78] conducted a study to investigate the behavior of nano/microplastics in wastewater and their removal using membrane processes, particularly the effectiveness of MOF-based membranes for NP/MP removal. In their study, they utilized MOF-based ED-MIL 101(Cr) UF membrane to remove nano/microplastics from wastewater. According to their findings, the MOF-based ED-MIL 101(Cr) UF membrane exhibited efficient water permeability and had the potential to remove over 90% of negatively and positively charged nano/microplastics from wastewater through electrostatic forces of attraction and repulsion. The research showed that MPs have the ability to break down into smaller particles in wastewater, which could lead to an increase in their distribution and harmful effects. This emphasizes the need to remove MPs from wastewater before they break down further and potentially contaminate the environment. Additionally, the study pointed out that MPs can interact with other pollutants in wastewater, like heavy metals and organic compounds. This interaction may change the behavior and toxicity of MPs, making their removal from wastewater even more crucial.

The effectiveness of various membrane processes in removing MPs from wastewater was studied by the researchers. They discovered that microfiltration and ultrafiltration membranes were successful in removing MPs, with removal rates ranging from 80 to 99%. However, they observed that nanofiltration and reverse osmosis membranes were not as efficient due to their smaller pore sizes, which can lead to fouling and reduced membrane performance over time.

They also examined how different operating parameters affected the removal of MPs using membrane processes. They discovered that increasing the transmembrane pressure and decreasing the cross-flow velocity improved the removal efficiency, but this also raised the likelihood of membrane fouling. Additionally, they observed that pretreating the wastewater to eliminate suspended solids and other pollutants can enhance membrane performance and the efficiency of MP removal.

In a recent research performed by Golgoli et al. [79], a thin film composite for forward osmosis membranes as a water-stable and hydrophilic metal–organic framework is developed by incorporation of various concentrations of MIL-53(Fe) as a prospective additive in substrate layer of polysulfone membranes. They systematically evaluated the membrane chemistry and its morphology which showed higher performance, antifouling behavior, hydrophilicity, roughness, and porosity in comparison to the untreated membrane. The optimized membranes (with 0.2 wt% MIL-53(Fe) loading) indicated a smoother and more hydrophilic surface having a more nodular structure. The presence of the MOF increased the porosity and hydrophilicity of the substrates leading to a higher water flux. This research revealed that the control membrane had a flux recovery of 73%, while the modified membrane had a full flux recovery only after it is physically cleaned.

MOFs with nanoscale pore sizes can still help remove microplastic particles despite the size mismatch. They can act as a barrier or sieve, preventing the passage of larger microplastics [77]. MOFs exhibit diverse surface properties, such as dual charge characteristics or specific functional groups, which enable them to effectively adsorb and capture microplastics [78]. The incorporation of MOFs into composite materials, such as foams and membranes, increases filtration performance by enhancing porosity and surface area, facilitating better contact between MOFs and contaminants. This leads to the effective removal of microplastics from water. Additionally, the use of MOFs increases substrate porosity and hydrophilicity, while forming a smooth and hydrophilic protective layer on the membrane surface, thereby improving antifouling properties [79]. Therefore, although MOFs may not perfectly match the pore size of microplastics, their integration into composites offers advantages such as size reduction, surface interactions, improved filtration performance, and enhanced antifouling properties, all of which contribute to the removal of microplastics from the aquatic environment.

As a result, the researchers reached the conclusion that traditional membranes can remove MPs but are not effective at removing nanoplastics, while MOF is capable of removing MPs by more than 90%. In general, the findings indicate that membrane processes have the potential to be a useful method for removing MPs from wastewater, but the effectiveness of these processes may vary depending on the type of membrane and operating parameters used. Further research is required to enhance the design of membranes and optimize operating conditions for MPs removal, as well as to better comprehend the interactions between MPs and other pollutants in wastewater.

In their research, Qin et al. [80] tried to tackle two significant environmental problems: the buildup of MPs and the necessity for renewable energy sources. Their proposed solution involves a new photocatalytic process that employs the Ag_2_O/Fe-MOF catalyst. This process can transform polyethylene glycol (PEG), PE, and polyethylene terephthalate (PET) plastics into useful chemicals, and additionally generate hydrogen. The photocatalytic and non-homogeneous photocatalysts are the basis of this approach, utilizing light to transform MPs into valuable substances. The scientists used a fresh approach to create photocatalysts for the conversion of MPs by transforming the metallic locations on a MOF into semi-conductive nanoparticles. The FeAg-MOF was the precursor used, and the exposure to light caused the formation of 6 nm Ag_2_O particles within the MOF structure. The resulting Ag_2_O/Fe-MOF has active sites that absorb a vast range of solar light, allowing it to be an efficient catalyst for converting MPs into useful chemicals and producing hydrogen. The study findings indicated that the Ag_2_O/Fe-MOF catalyst was more effective in terms of its photocatalytic efficiency and hydrogen production rate when compared to pure Ag_2_O. Additionally, it exhibited selectivity in converting MPs into useful chemicals, presenting a sustainable and eco-friendly solution for managing plastic waste. This innovative chemical synthesis technique could be a potential solution for managing plastic waste sustainably and producing hydrogen, thus reducing the adverse environmental effects of plastic waste and promoting the utilization of renewable energy resources. They have proposed a hopeful resolution to tackle the environmental concern of MP waste. The technique they proposed involves transforming the metallic sites on MOFs into semiconductive particles that work as photocatalysts to convert MPs into valuable substances. The photocatalytic process exploits light energy, which stimulates the conversion of MPs into useful products.

To sum up, the method of forming Ag_2_O in a MOF to upcycle MPs and produce hydrogen via light exposure holds great promise as a solution for managing plastic waste. The Ag_2_O/Fe-MOF photocatalysts demonstrate excellent photocatalytic efficiency and hydrogen production rates, offering a fresh and sustainable method for plastic waste management. The outcomes of this study could potentially advance the development of practical and eco-friendly techniques for managing plastic waste and generating hydrogen.

The method utilized by the researchers, which involves the creation of semi-conductive particles within MOFs, offers a simple way to upcycle MPs and generate hydrogen. The outcome is a heterojunction photocatalyst with a wide range of light absorption and enhanced charge transfer rate. This method presents a hopeful approach in tackling one of the most pressing environmental problems of our time and lays the groundwork for more effective and sustainable management of plastic waste.

You et al. [81] used MOF-based wood aerogel for removing micro/nano plastics. The results of the study indicate that ZIF-8@Aerogel has a highly favorable effect on the removal of nano-scale PVDF and PS particles. The removal efficiencies for these particles reached 91.4% and 85.8%, respectively. In comparison, a similar experiment was conducted using only aerogel to remove the MPs, and the removal efficiencies were found to be significantly lower, at 56.9% for PVDF and 42.5% for PS. To further verify the removal performance of ZIF-8@Aerogel in natural aquatic environments, the researchers conducted a similar experiment using seawater to simulate a MP suspension. The results showed that the removal efficiency of ZIF-8@Aerogel on PVDF and PS in seawater was similar to that in water, with efficiencies of 92.5% and 88.7%, respectively. This demonstrates the potential of ZIF-8@Aerogel to effectively remove nano-scale MPs in real-world applications. In conclusion, the results of the study demonstrate the favorable removal effect of ZIF-8@Aerogel on nano-scale PVDF and PS particles, both in water and in a simulated seawater environment. The high removal efficiencies achieved by ZIF-8@Aerogel make it a promising solution for removing MPs in aquatic environments.

The removal performance of ZIF-8@Aerogel on MPs is the result of a combination of different factors. The first factor is the strong electrostatic interaction between the positively charged ZIF-8 within the aerogel and the negatively charged MP particles. This interaction is due to the difference in charge between the two materials, which attracts the MPs to the ZIF-8. Another factor is the hydrophobic interaction between the MPs and ZIF-8. This interaction is enhanced by the fact that both PVDF, PS, and ZIF-8 are hydrophobic substances, meaning they repel water. This hydrophobic interaction makes it easier for the ZIF-8 to trap and remove the MPs from the water. The study also found that hydrogen bonds are formed between the -OH in aerogel cellulose and C-F in PVDF, contributing to the removal performance of the composite material. Additionally, there is a potential van der Waals force between the MP molecules and the composite material molecules. These combined effects result in the favorable performance of ZIF-8@Aerogel in removing MPs from water.

In summary, the removal performance of ZIF-8@Aerogel on MPs is due to a combination of electrostatic interactions, hydrophobic interactions, hydrogen bonds, and van der Waals forces. These factors work together to enhance the ability of the composite material to effectively remove MPs from water.

Pasanen et al. [20] conducted a study to assess the effectiveness of nano-Fe@ZIF-8 in removing MP. The researchers used 1.1 μm PS microspheres as a representation of environmental MPs in the low size range. To prevent instability during the MP removal process, the experiments were performed at a reduced mixer speed of 700 rpm. The researchers found that using 5 mg of nano-Fe@ZIF-8 and an agitation time of 3 min, the relative standard deviation for three consecutive MP removals was 17%. The study showed that nano-Fe@ZIF-8 (1:4) was a successful sorbent for removing MPs, with higher removal efficiencies compared to the larger Fe@ZIF-8 particles. This improvement was due to the smaller particle size, which increased the available effective surface area for interaction between the ZIF crystals and the PS microspheres.

The study also examined the effect of agitation time on the MP removal efficiency and found that the removal efficiency increased with an increase in agitation time, reaching a maximum at 5 min. The researchers found that optimal removal was achieved using 20 mg of nano-Fe@ZIF-8 (1:4) for 25 mg/L samples and 30 mg for 50 mg/L samples. The researchers also found that the nano-Fe@ZIF-8 was effective in removing MPs with different sizes and functionalities, including 15 μm PS beads and 1 μm carboxyl-functionalized PS beads. Additionally, the researchers found that the nano-Fe@ZIF-8 was capable of removing both bisphenol A and 4-tert-butylphenol, endocrine-disrupting phenols, without decreasing its efficiency in removing the PS microspheres. In this study, the effect of Fe@ZIF-8 particle size on the removal of PS microspheres was analyzed. The results showed that the smaller the Fe@ZIF-8 particle size, the more effective the removal of MPs. The nano-Fe@ZIF-8 (1:4) showed the highest removal efficiency, with 65.4 ± 5.1% of 25 mg/L PS microspheres removed, increasing to 81.2 ± 5.6% with nano-Fe@ZIF-8 (1:4). A similar trend was observed for higher concentrations of PS microspheres, with nano-Fe@ZIF-8 (1:4) removing 73.0 ± 3.3% while Fe@ZIF-8 only removed 40.9 ± 2.6%. The improvement in removal efficiency was attributed to the increase in effective surface area of interaction between the ZIF crystals and the PS microspheres as a result of the decrease in ZIF particle size.

The effect of agitation time was also studied, with the removal efficiency increasing with time and reaching an optimum at 5 min, with 77.0 ± 1.7% and 82.3 ± 2.4% of MPs removed for 25 mg/L and 50 mg/L MPs, respectively. The optimal removal of MPs was achieved using 20 mg of nano-Fe@ZIF-8 (1:4) for 25 mg/L PS microspheres, with 98.0 ± 0.2% removal within 5 min while the optimal removal of 50 mg/L PS microspheres was obtained with 30 mg of nano-Fe@ZIF-8 (1:4), with 88.7 ± 1.5% removal within 5 min.

The FT-IR results showed no significant changes in the vibrations of nano-Fe@ZIF-8 before and after the removal of PS microspheres, confirming its viability for MP removal. The results were also confirmed for different sizes and functionalities of PS microspheres, with 96.5 ± 1.0% removal efficiency observed for 15 μm PS beads and 82.0 ± 2.6% removal efficiency observed for 1 μm carboxyl functionalized PS beads.

Nano-Fe@ZIF-8 (1:4) was also found to be effective in the simultaneous removal of endocrine disrupting phenols, Bisphenol A, and 4-tert-butylphenol, with 97.6 ± 0.6% and 95.8 ± 0.5% removal, respectively, and a slightly lower but still above 90% removal of MPs. The results were also confirmed for real water sample matrices, with no significant changes in removal efficiency compared to results obtained with distilled water.

**Fig. 3:**
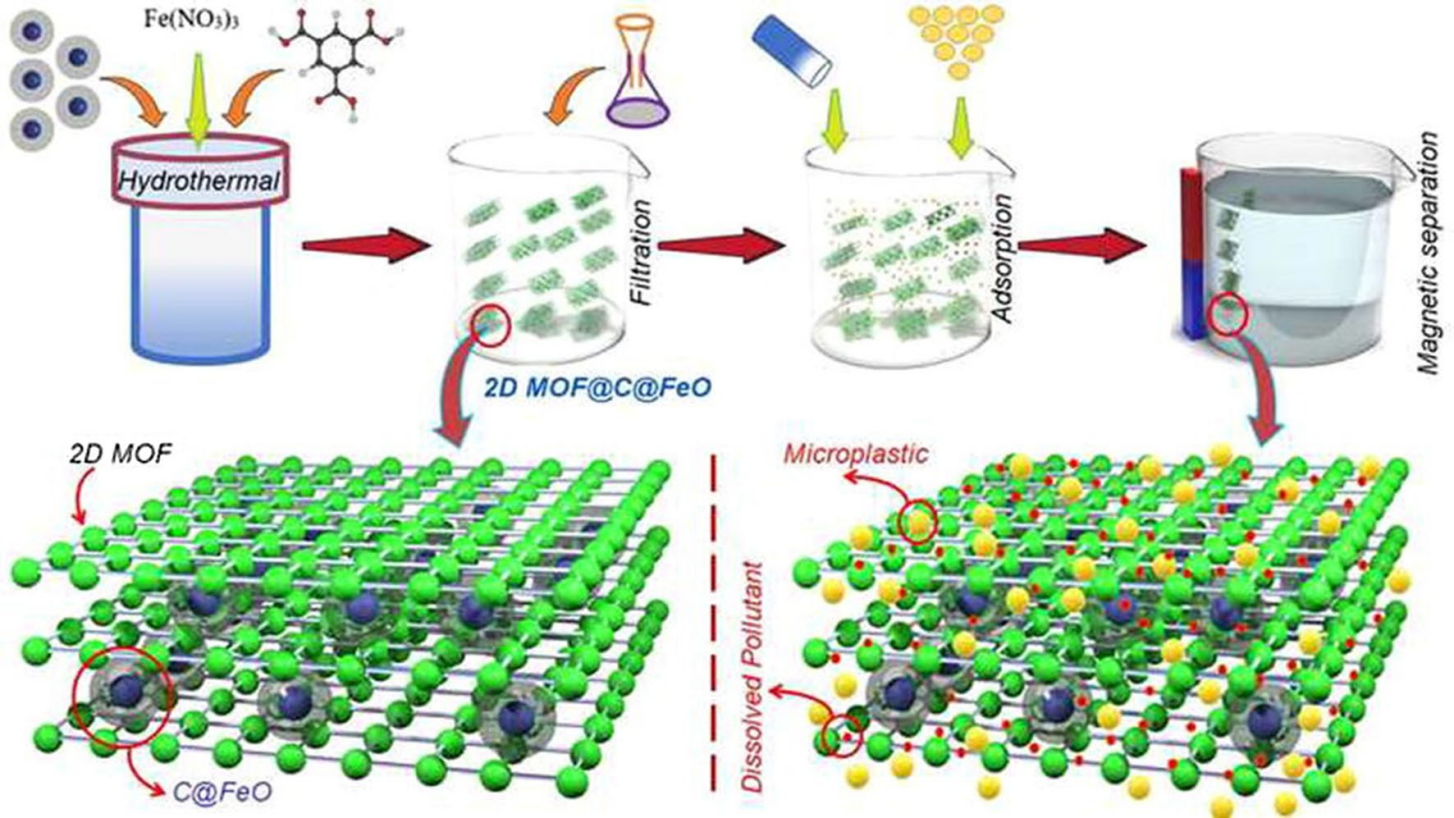
The structure of a 2D MOF@C@FeO nanopillared with adsorption process, magnetic separation, and MPs removal pathway. MOF sheets are separated using carbon encapsulated iron oxide. Carbon have strong connection with MOF sheets to stabilize them and magnetic core helps easy separation (From [19] with kind permission of the copyright owner)

Since traditional adsorbents are not adequate to remove MP due to various technical and structural challenges Haris et al. [19] created a new method for removing both solid and dissolved contaminants from water using a nanopillared structure made up of a two-dimensional MOF and carbon encapsulated iron oxide (C@FeO) nanopillars (Fig. 3). The structure boasted a large surface area of 749.7 m^2^/g, many active sites, and magnetic properties that make it easy to separate pollutants from the water.

To address these issues, a new 2D nanopillared heterostructure was developed by growing a 2D MOF on magnetic nanoparticles made of C@FeO. The self-assembly of C@FeO nanopillars between the MOF sheets prevents the 2D sheets from sticking together, thereby significantly increasing the surface area. Additionally, the magnetic properties of C@FeO nanopillars make it easy to separate the adsorbent from the water. C@FeO can remove 71.7% of MP with concentration of 1000 mg/L in 60 min while the 2D MOF@C@FeO was found to remove 100% of both MP and MB from a binary system in just 60 min. In a single system, 60 and 30 min were required to remove MP and MB, respectively.

However, pure 2D MOF poses a challenge in separation and cannot be used without additional filtration or centrifugation. The self-assembly of C@FeO nanopillars between the MOF sheets enhances the surface area and allows for easy separation of the adsorbent from the water using an external magnet. This not only makes the system more convenient to use but also reduces material use and operational cost. The results of the study showed that the 2D nanopillared heterostructure, developed by in situ growth of a 2D MOF on magnetic nanoparticles made of C@FeO, is an effective adsorbent for the removal of both MP and MB from water.

The 2D MOF@C@FeO was tested for reusability and stability over six consecutive adsorption cycles and removal efficiency was obtained 90%. The results of this study were supported by ex-situ characterization, which showed that the materials maintained their stability after multiple cycles. This proves that 2D MOF@C@FeO is a reliable and sustainable solution for the removal of contaminants from water.

Overall, the results demonstrated that 2D MOF@C@FeO is a superior alternative to conventional adsorbents and traditional filtration methods due to its high adsorption capacity, magnetic removal, and easy use. The results of this study can have important implications for the water treatment industry, as it provides a new and effective solution for removing contaminants from water, particularly MP and MB.

Wan et al. [38] conducted a study to investigate the effectiveness of using ZIF-67, a type of MOF material, for extracting MPs from water-based solutions. Their objective was to explore the potential of MOF materials for treating wastewater contaminated with MPs. The researchers observed that the adsorption of polystyrene microplastics (PSMPs) by ZIF-67 increased with the increase in the dose of ZIF-67 from 0.1 to 0.6 g/L, with an adsorption rate rising from 65.4 to 90.2%. This can be attributed to the availability of more adsorption sites as the dose of ZIF-67 increases. However, when the dose of ZIF-67 exceeded 0.4 g/L, the adsorption ratio remained largely constant. Moreover, as the dose of ZIF-67 increased, the amount of PSMPs being adsorbed decreased from 34.5 to 7.2 mg/g. The optimal dose for the removal of PSMPs was determined to be 0.4 g/L of ZIF-67, with an adsorption rate of 92.1% and an adsorption capacity of 11.6 mg/g. It is therefore recommended to use 0.4 g/L of ZIF-67 for the effective removal of PSMPs.

Within a pH range of 3 to 10, ZIF-67 was observed to maintain its ability to adsorb PSMPs at a rate of approximately 88.3%. This high rate of PSMPs adsorption was primarily due to the strong electrostatic attraction between positively charged ZIF-67 and negatively charged PSMPs. Additionally, the pi-pi stacking and hydrogen bonding between ZIF-67 and PSMPs were likely contributing factors to the effective removal of PSMPs from the aqueous solution. However, at highly alkaline conditions (pH range of 11 to 12), the negatively charged ZIF-67 and negatively charged PSMPs experienced a repulsive force, leading to a decrease in the removal ratio of PSMPs by ZIF-67 to 64.4%. As such, it is recommended that ZIF-67 be used for the adsorption of PSMPs within a pH range of 3 to 10, with the highest rate of PSMPs adsorption observed at a pH around 8.

The researchers found that when the temperature was changed from 288 to 308 K, the PSMP adsorption rate by ZIF-67 initially increased slightly, from 83.1 to 92.1%, and then decreased to 86.4%. The increase in adsorption capacity, from 10.3 mg/g to 11.5 mg/g, when the temperature increased from 288 to 298 K, was due to physical adsorption being the primary driving force behind PSMPs adsorption by ZIF-67, especially at lower temperatures. At 298 K, chemical adsorption became the primary force behind PSMPs adsorption, and the number of active adsorption sites increased with increasing temperature. The slight decrease in adsorption capacity, from 11.5 to 10.6 mg/g, may have been due to the desorption of PSMPs from ZIF-67 due to the increased thermal motion of particles with rising temperature. These findings suggest that increasing temperature negatively affects the removal of MPs by MOF materials in aqueous solutions. Therefore, the Freundlich model constants were not calculated at various temperatures, and the thermodynamic results were not discussed any further.

To summarize, the study found that ZIF-67 is a promising material for removing MPs from water, thanks to its effective use of hydrogen bonding, electrostatic attraction, and π–π stacking. These findings have the potential to contribute to the development of new wastewater treatment methods for removing MPs, especially in environments with low to moderate alkalinity.

## Conclusions

So far, there is no specific technique to entirely remove MPs from aqueous environment [82]. Geometry, size and density of them dictate how they can be dispersed. Large, dense and irregular-shaped particles tend to sediment underwater while smaller, lighter and spherical-shaped MPs are retained on the surface. MOFs have emerged as a promising solution for removing MPs from aqueous environments due to their high surface area, tailored porosity, renewability, chemical stability, and versatility. Although MOFs have some drawbacks, such as their often-unstable powder form, these can be overcome by supporting MOFs on other substrates, such as aerogels or foams.

As the issue of MP pollution continues to grow, it is essential to develop effective and sustainable methods for removing them from aquatic ecosystems. MOFs show great potential as a solution to this problem and could be integrated into existing water treatment systems in the future.

However, more research is needed to explore the practical implementation of MOFs for MP removal on a larger scale, including their cost-effectiveness and scalability. Nonetheless, MOFs represent a promising avenue for developing innovative solutions to address the ongoing issue of MPs pollution in our environment.

Here are some suggestions for further research and development on the use of MOFs for MPs removal from aqueous environments:

- Optimization of MOF properties: Researchers can investigate ways to optimize the properties of MOFs, such as their pore size, surface area, and chemical composition, to enhance their efficiency in removing MPs. For example, they can design MOFs with larger pore sizes to adsorb larger MPs particles, or modify the chemical composition of MOFs to enhance their affinity for certain types of MPs.
- Integration with existing water treatment systems: MOFs can be integrated into existing water treatment systems, such as sand filters or activated carbon filters, to enhance their MP removal capacity. This can be achieved by adding MOFs as an additional layer of filtration, or by replacing existing filter media with MOFs.
- Scale-up production: Scaling up MOF production can reduce their cost and make them more accessible for widespread use in MP removal applications. This can be achieved through process optimization, improving the yield of MOF synthesis, and exploring new methods for large-scale MOF production.
- Long-term stability and regeneration: More research is needed to investigate the long-term stability of MOFs in aqueous environments and develop efficient regeneration methods for their repeated use. This can include investigating the effects of water chemistry and temperature on MOF stability, and exploring regeneration methods such as heating or washing to ensure the long-term effectiveness of MOFs.
- Real-world application: Testing MOFs under realistic conditions, including variable water chemistry and flow rates, can help to assess their effectiveness and identify any potential limitations. This can involve testing MOFs in different aquatic environments, such as rivers or lakes, and under different flow rates to ensure their effectiveness in removing MPs.

Overall, these suggestions can help to advance the use of MOFs as a sustainable and effective solution for MP removal from aqueous environments, and provide important insights into their practical application in real-world settings. A Scopus search reveals that in comparison to 2012, in 2022, 7.7 times more articles related to MOF were published which shows the importance of these unique compounds in the industries and daily life during the following years. Access to clean water is still a major problem for a considerable percentage of the world’s population which is predicted to grow to 8.5 billion by 2030 and 9.7 billion by 2050. Climate change and Long periods of drought affect clean water supplies, while contaminants such as MPs can pollute clean water sources and cause outbreaks of disease. Utilizing MOFs in water refineries can greatly help in supplying clean water for the increasing population.

### Supplementary Information

**Additional file 1: Table S1.** Conventional methods for removal of MPs. From [34] with kind permission of the copyright owner.

## Data Availability

All data generated or analyzed during this study are included in this published article.

## Abbreviations

HDPE: High-density polyethylene
PVC: Polyvinyl chloride
PS: Polystyrene
PP: Polypropylen
PES: Polyethersulfone
PVDF: Polyvinylidene fluoride
PMMA: Polymethyl methacrylate
PEG: Polyethylene glycol
PE: Polyethylene
PET: Polyethylene terephthalate
PSMPs: Polystyrene microplastics
MPs: Microplastics
MOFs: Metal–organic frameworks
