# Supplementary material for: Microplastics removal from aqueous environment by metal organic frameworks
Source: BMC Chem. 2023 Sep 21;17(1):122. doi: 10.1186/s13065-023-01032-y (PMC10514943; doi:10.1186/s13065-023-01032-y)
Supplement: Supplementary file 1 — Additional file 1: Table S1. Conventional methods for removal of MPs. From [34] with kind permission of the copyright owner. [file 13065_2023_1032_MOESM1_ESM.docx]

**Additional material to:**

**Microplastics removal from aqueous environment by metal organic frameworks**

Zhila Honarmandrad, Massoud Kaykhaii^*^ and Jacek Gębicki

*Department of Process Engineering and Chemical Technology, Faculty of Chemistry, Gdańsk University of Technology, Narutowicza 11/12, Gdańsk 80-233, Poland*

*Corresponding author. E-mail: massoud.kaykhaii@pg.edu.pl; Tel: +48731960312

**Table S1.** Conventional methods for removal of MPs. From [34] with kind permission of the copyright owner.

| **Process description** | **Major mechanism** | **Lowest size of microplastic particle removed/finest mesh** | **Efficiency (%)** | **Advantages** | **Challenges** |  |
| --- | --- | --- | --- | --- | --- | --- |
| Wastewater treatment plant processes | Skimming, settling of the entrapped microplastics | 300 μm | 99.9 | Conventional process, no additional cost | Not possible to remove MPs of size <300 μm |  |
|  | Primary, secondary, and tertiary | 100 μm | 99.9 | Conventional process, no additional cost | Not possible to remove MPs of size <100 μm |  |
|  | Secondary treatment | 20 μm | 95.6 | MBR process exhibited greater overall efficiency | Complete retention is not possible |  |
|  | Tertiary treatment |  | 97.2 |  |  |  |
|  | Membrane bioreactor |  | 99.4 |  |  |  |
|  | Membrane bioreactor (MBR) | 250 μm | 99.3 | MBR process helped to retain more microplastics compared to conventional activated sludge process | Not possible to remove MPs of size <250 μm |  |
| Al and Fe salts | Coagulation | <0.5 mm | 45.34±3.93 | Simple process, does not require additional set-up | Low efficiency |  |
| Electrocoagulation | Charge neutralization, flocculation | – | 90 (pH 3–10) 99.24 (pH 7.5) | Does not rely on chemicals or microorganisms, energy efficient | Operation time needs to lowered down |  |
| Filtration with biochar | Morphologically controlled mechanism (Stuck, trapped, entangled) | 10 μm | >95 | Low cost and efficient | Process is slow and results in obstruction of the pores with time; costly; regeneration is tough |  |
| Filtration with granular activated carbon (combined with coagulation and sedimentation) | Physical properties (size and shape) | 1–5 μm | 56.8–60.9 | Efficient to remove plastic particles of nano-size range | Process is slow and results in obstruction of the pores with time; costly; regeneration is tough |  |
| Pulse clarification with filtration | Entrapment in sludge blanket formed due to coagulation floats | <100 μm | 85 | Removal efficiency is comparable to the other treatment plants having advanced processes | Complete retention is not possible |  |
| Algal masses | Electrostatic charge onto the microplastic particles and algal surfaces | 20 μm | 94.5 | No chemical, electrical, and mechanical operations | Efficiency will vary owing to physiological and topographical differences on the seaweed surface |  |
| Bioinspired molecules | Mechanical capture mechanism driven by the hydrophobic and van der Waals interactions | – | – | Flexible; possibility to remove different types of plastic particles in wastewater stream | Method yet to be established for practical purposes |  |
| Zr metal organic framework (MOF)-based foams | Entrapment | – | 95.5±1.2 | High performance, excellent durability | Flexibility and robustness of the MOF-based foams; Removal efficiency affected by the particle size and zeta potential |  |
| Photocatalytic micromotors | Phoretic interaction and shovelling/pushing interactions | – | – | Self-propelled devices, works efficiently independent of the fuel | Selectivity of micromotors for microplastics is crucial |  |
